# Recurrent refractory Kawasaki disease

**DOI:** 10.1186/1546-0096-9-S1-P98

**Published:** 2011-09-14

**Authors:** P Costa Reis, S Nativ, A Starr, L Imundo, A Eichenfield

**Affiliations:** 1 Department of Pediatrics, Santa Maria Hospital, Lisbon, Portugal; 2 Division of Rheumatology, Morgan Stanley Children’s Hospital of New York-Presbyterian, Columbia University Medical Center (MSCHONY/CUMC), New York, USA

## Background

Kawasaki disease is a common childhood vasculitis. Unrelenting fever after treatment with intravenous immunoglobulin (IVIG) occurs in 10-15% of patients and is associated with a greater risk of developing coronary aneurysms.

## Aim

Describe a very unique case of recurrent and refractory Kawasaki disease.

## Case report

A 3 year old boy presented with 3 days of fever, rash, pharyngeal and gingival erythema, and swollen extremities. Laboratory investigations revealed leukocytosis, C–reactive protein 25.8 mg/dl, and erythrocyte sedimentation rate 100 mm/hr. Echocardiography disclosed diffuse dilatation of all proximal coronary arteries. The child received IVIG (2g/kg) and aspirin (100 mg/kg/d) with no response. IVIG was repeated, followed by methylprednisolone 30 mg/kg for 3 days, but the child remained febrile. Infliximab (5 mg/kg) was thereupon employed with prompt defervescence. Low-dose aspirin was continued, as well as clopidogrel. Echocardiographic findings remained stable.

Six months after the initial episode, the child again presented with fever, irritability, sore throat and nuchal rigidity. Physical examination revealed cracked, swollen lips, oropharyngeal erythema, posterior cervical lymphadenopathy, and rash. Desquamation of the distal extremities was observed some days later. Aneurysms were detected, involving the left and right main coronary arteries, as well as the left anterior descending coronary. Magnetic resonance angiography of the chest and abdomen revealed no other involved vessels. The child again received IVIG, pulse methylprednisolone, and infliximab, but remained febrile and developed significant arthritis, requiring daily prednisolone. He is now asymptomatic.

## Conclusions

Currently, recurrent and refractory Kawasaki disease still represents a therapeutic challenge.

